# AER-DCWGAN: Adversarial Encoder-Regularized Dual-Conditional Wasserstein GAN for Imbalanced Network Intrusion Detection

**DOI:** 10.3390/s26144506

**Published:** 2026-07-15

**Authors:** Mingqi Wang, Yu Yang, Minna Gao, Jinliang Yuan

**Affiliations:** 1 School of Information Engineering, Engineering University of PAP, Xi’an 710086, China; wangmq202412@163.com (M.W.); lighteryuan@163.com (J.Y.); 2 College of Missile Engineering, Rocket Military Engineering University, Xi’an 710086, China; gmn0127@sina.com

**Keywords:** network intrusion detection, class imbalance, data augmentation, Wasserstein GAN, adversarial encoder regularization, minority attack synthesis

## Abstract

Class imbalance remains a major obstacle to reliable network intrusion detection, particularly in Internet of Things (IoT) and sensor-network monitoring scenarios where rare attack categories are represented by only a small number of high-dimensional traffic samples. To improve minority-class augmentation, we propose an adversarial encoder-regularized dual-conditional Wasserstein generative adversarial network (AER-DCWGAN), a class-aware latent-consistency framework operating in a normalized, feature-selected space. Unlike label-only conditional generation, AER-DCWGAN jointly models traffic features, latent codes, and class embeddings and is designed to encourage feature-, latent-, and label-conditioned consistency. The framework integrates a latent-code- and label-aware Wasserstein critic, encoder-guided reconstruction, adversarial prior alignment, and label-consistency filtering to reduce latent drifting and suppress semantically ambiguous generated samples. Experiments on NSL-KDD and CIC-IDS2017 show class-dependent effects rather than uniform improvement. On NSL-KDD, the Remote-to-Local (R2L) F1-score increases from 0.501 to 0.823, whereas the User-to-Root (U2R) F1-score increases only from 0.124 to 0.204 with a recall of 0.270, indicating that U2R detection remains weak. On CIC-IDS2017, Web Attack improves from 0.952 to 0.983, but Bot and PortScan decrease slightly from 0.828 to 0.817 and from 0.996 to 0.994, respectively. The improvement reported for Infiltration should also be interpreted cautiously because the test support is only seven samples. The controlled head-to-head comparison is restricted to the closely related WGAN-GP and AE-WGAN baselines, and the generated samples are evaluated and used only in the processed feature space; therefore, the study does not claim broad superiority over all imbalance-handling strategies or protocol-level validity of reconstructed raw traffic. Overall, AER-DCWGAN alleviates moderate class imbalance for several classes with sufficient representation, but it does not fully solve ultra-rare attack detection.

## 1. Introduction

With the rapid advancement of the Internet of Everything (IoE), network security has evolved from a technical challenge into a strategic concern affecting national security, economic operations, and social stability. As the frequency and impact of network attacks continue to increase, reducing the damage caused by malicious traffic has become increasingly urgent [1]. Network intrusion detection systems (NIDS), as core components of active defense frameworks, play an important role in monitoring malicious activities, unauthorized access, and potential threats in real time [2,3]. However, NIDS still face a critical deployment challenge: extreme class imbalance. For example, in the standard KDDTrain+ split of NSL-KDD, normal traffic accounts for 67,343 of 125,973 records (53.46%), whereas the User-to-Root (U2R) class contains only 52 records (0.04%) [4]. Such imbalanced biases classifiers toward majority classes and weakens their ability to identify rare but security-critical attacks.

To address class imbalance, traditional data balancing methods—including random undersampling [5], random oversampling [6], Synthetic Minority Over-sampling Technique (SMOTE) [7], and Adaptive Synthetic Sampling (ADASYN) [8]—have been widely adopted. These methods can increase minority-class representation, but they also introduce important limitations, such as majority-class information loss, repeated or noisy samples, and possible data leakage. More importantly, interpolation-based oversampling is often insufficient for high-dimensional network traffic, where protocol, service, duration, and flow-statistical features interact nonlinearly. In recent years, deep generative augmentation methods, especially Generative Adversarial Networks (GANs) [9], have been explored to learn minority-class attack distributions and synthesize additional training samples.

Despite this progress, three limitations remain insufficiently addressed in intrusion-detection data augmentation. First, conventional oversampling methods operate mainly in the original feature space and rely on local interpolation assumptions, which may distort nonlinear traffic-feature relationships. Second, many conditional GAN variants inject class labels only as auxiliary inputs; as a result, the discriminator may not remain sufficiently sensitive to class-conditioned boundaries during distribution alignment, and generated samples can become statistically plausible but semantically ambiguous. Third, encoder–generator hybrids may improve feature representation, but without explicit latent-space regularization, they can suffer from latent drifting, unstable generation, and reduced diversity for minority attacks.

To address these issues, we propose AER-DCWGAN, an adversarial encoder-regularized dual-conditional Wasserstein framework for minority-class traffic synthesis. The central idea is to formulate data augmentation not as label-conditioned sample generation alone, but as class-aware latent-consistency learning over three coupled spaces: the normalized and feature-selected traffic space, the latent-code space, and the class-semantic embedding space. In this formulation, generated samples are optimized for compatibility with the processed feature distribution, latent codes, and class labels under which they are synthesized. Therefore, the proposed method aims to reduce class-boundary ambiguity and latent-space discontinuity for minority classes with sufficient training support; it is not intended to establish protocol-level validity for raw traffic records.

The contribution statement is consolidated into three core points to avoid presenting standard components as independent innovations:

- We propose AER-DCWGAN, a dual-conditional Wasserstein augmentation framework that explicitly incorporates feature–latent–label tuple consistency into the critic scoring process for imbalanced NIDS data. Instead of treating the feature–latent–label consistency issue as a separate contribution, it is defined as the central design principle of the framework: the critic evaluates real tuples (E(x),x,y) and generated tuples (z,G(z,y),y) so that feature realism, latent-source consistency, and class semantics are considered jointly.
- We adapt adversarial encoder regularization (AER) to align the encoder-induced latent distribution with the same Gaussian prior used by the generator. Although adversarial prior matching is related to adversarial autoencoder-style regularization, its role here is specific: it reduces latent-source artifacts between encoded real samples and randomly sampled generator inputs, thereby mitigating latent-space drift in the dual-conditional WGAN training process.
- We construct a leakage-free offline augmentation–detection pipeline and evaluate it under controlled experimental settings on NSL-KDD and CIC-IDS2017. The revised protocol keeps preprocessing, feature selection, augmentation, validation, downstream classifier training, and test evaluation separated. The controlled head-to-head comparison is deliberately restricted to the closely related WGAN-GP and AE-WGAN baselines; SMOTE, ADASYN, CTGAN, TVAE, and other imbalance-handling strategies are not included in this controlled performance benchmark, so no broad superiority claim is made.

## 2. Related Work

### 2.1. Imbalanced Intrusion Detection and Conventional Balancing

Network intrusion detection datasets are typically long-tailed: normal traffic and common attacks dominate the training distribution, whereas critical attack categories such as User-to-Root (U2R), Remote-to-Local (R2L), Bot, Infiltration, and Web attacks may contain only a small number of samples. For example, the standard NSL-KDD split contains only 52 U2R records in KDDTrain+, which makes minority-class detection highly sensitive to sampling strategy and classifier bias [4]. Conventional imbalance-handling methods are usually divided into data-level and algorithm-level approaches. Data-level methods include random undersampling, random oversampling, SMOTE, ADASYN, and hybrid strategies [10,11,12]. Algorithm-level methods include cost-sensitive learning, boosting, and class-weighted objectives [13,14]. Although these methods are simple and efficient, interpolation-based oversampling can introduce unrealistically high-dimensional flow features, while undersampling may discard informative majority-class patterns. They also do not explicitly learn the latent distribution of minority attacks.

### 2.2. Deep Learning-Based Intrusion Detection

Deep learning methods have improved intrusion detection by learning nonlinear feature representations from network traffic. Convolutional Neural Network (CNN)-based, attention-based, ensemble, and hybrid models have been used to reduce manual feature engineering and improve generalization [15,16,17,18,19]. However, classifier-side improvements alone cannot fully address severe data imbalance. When minority classes are extremely sparse, the learned decision boundary may still be dominated by majority-class samples, and high overall accuracy may hide poor recall for rare attacks. This motivates data augmentation methods that not only rebalance labels but also preserve realistic traffic-feature semantics.

### 2.3. GAN-Based Data Augmentation for Minority Attacks

Generative Adversarial Networks (GANs) have become an important tool for imbalanced intrusion detection because they can model nonlinear data distributions beyond simple interpolation. Early conditional GAN-based models attempted to generate minority attack samples with class information [20,21]. Wasserstein-based variants further improved training stability and sample quality by replacing the original adversarial divergence with the Wasserstein distance and, in some cases, gradient penalty [22,23,24]. Hybrid Autoencoder (AE)/Variational Autoencoder (VAE)–GAN models introduce reconstruction constraints or latent representations to improve sample fidelity [25,26]. Recent work also shows two important trends. Kang et al. [27] proposed an M2M-VAEGAN framework that combines Variational Gaussian Mixture (VGM) preprocessing, majority-class pre-training, balanced fine-tuning, and an auxiliary classifier to improve cross-class minority generation on NSL-KDD and CIC-IDS2017. Li et al. [28] integrated Soft Nearest Neighbor Loss (SNNL) into Wasserstein GAN (WGAN), Conditional WGAN (CWGAN), and WGAN-GP, showing that explicit feature-space regularization can improve minority-class F1-scores on NSL-KDD, CSE-CIC-IDS2017, and CSE-CIC-IDS2018 while adding moderate training overhead. These studies confirm that recent GAN-based augmentation is moving from simple sample synthesis toward class-aware, feature-aligned, and deployment-conscious generation.

Despite these advances, three issues remain relevant to the present work. First, conditional generation may still produce samples that match the target label superficially but are inconsistent with the latent source distribution. Second, AE/VAE–GAN frameworks often use reconstruction or latent mapping but do not always adversarially align the encoder output with the generator prior. Third, feature-space regularization and auxiliary classification improve semantic consistency, yet many methods do not jointly evaluate feature, label, and latent-code consistency within the critic.

### 2.4. Positioning of AER-DCWGAN

The proposed AER-DCWGAN is positioned as a task-specific augmentation framework for imbalanced network intrusion detection. Its novelty is not the isolated use of conditional labels, Wasserstein loss, gradient penalty, autoencoding, or reconstruction loss; rather, it lies in coordinating these components to address latent–feature–label inconsistency in minority-class traffic synthesis (As shown in Table 1). Compared with WGAN-GP and CWGAN-GP, AER-DCWGAN introduces an encoder-guided latent pathway and reconstruction regularization. Compared with AE-WGAN, the encoder is adversarially regularized toward the same Gaussian prior used by the generator, and the critic evaluates latent–feature–label tuples instead of generated features alone. Compared with Bidirectional Generative Adversarial Network (BiGAN)/enhanced BiGAN (E-BiGAN)-style models, the objective is not general bidirectional representation learning but label-consistent minority attack augmentation for downstream intrusion detectors.

## 3. Methodology

### 3.1. AER-DCWGAN Framework

**Figure 1:**
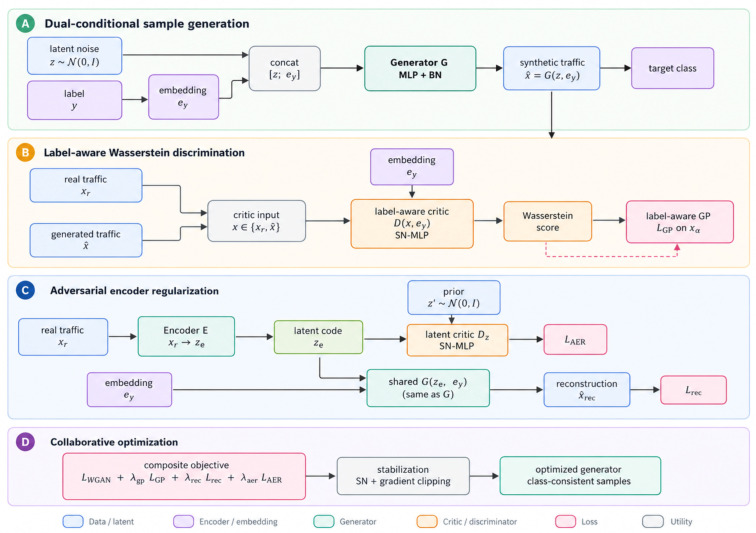
Overall architecture of AER-DCWGAN.

The proposed AER-DCWGAN is designed to generate class-conditioned minority samples in the normalized, feature-selected representation used by the downstream detectors. Instead of presenting GAN, Conditional GAN (CGAN), and WGAN as separate preliminary models, we directly build the formulation on the conditional Wasserstein objective with gradient penalty (GP), following the WGAN line of work [22,29]. Given a traffic sample $x\in R^{d_{x}}$, its class label *y*, and a latent variable z∼N(0,I), the model consists of four trainable components: an encoder *E*, a conditional generator *G*, a latent-code- and label-aware Wasserstein critic *D*, and a latent-space discriminator $D_{z}$. The overall architecture is shown in Figure 1.

#### 3.1.1. Dual-Conditional Generator

The class label *y* is first mapped to a learnable embedding vector $e_{y}=\mathrm{Emb}\left(y\right)\in R^{d_{e}}$. The generator takes the concatenation of the Gaussian noise and the label embedding as input and produces a synthetic processed feature vector conditioned on the target attack category. Specifically, $\hat{x}=G\left(z,e_{y}\right)=\tanh\left(f_{G}\left(\left[z;e_{y}\right]\right)\right)$, where z∼N(0,I) and $f_{G}\left(\cdot \right)$ denotes a multilayer perceptron with batch normalization. This design explicitly couples stochastic variation with class semantics, thereby enabling category-oriented synthesis for minority attacks such as R2L, U2R, Bot, and Infiltration.

#### 3.1.2. Latent-Code- and Label-Aware Wasserstein Critic

The critic receives a traffic sample, its associated latent code, and the corresponding label embedding and outputs a real-valued Wasserstein score rather than a binary probability. For a real sample, the associated latent code is obtained from the encoder, $z_{e}=E\left(x_{r}\right)$. For a generated sample, the associated latent code is the Gaussian noise *z* used by the generator. Thus, the critic can be written as $D\left(z_{x},x,e_{y}\right)=f_{D}\left(\left[z_{x};x;e_{y}\right]\right)$, where $\left(z_{x},x\right)\in \left\{\left(z_{e},x_{r}\right),\left(z,\hat{x}\right)\right\}$. This formulation makes the critic sensitive not only to feature-level realism and class semantics but also to the consistency between the latent code and the generated or reconstructed traffic representation. To strengthen class-conditioned distribution matching, the gradient penalty is computed under the same label condition. For $x_{\alpha }=\alpha x_{r}+\left(1-\alpha \right)\hat{x}$ and $z_{\alpha }=\alpha z_{e}+\left(1-\alpha \right)z$, with α∼U(0,1), the label-aware gradient penalty is defined as

(1) $$L_{\mathrm{GP}}=E_{\alpha ,x_{r},\hat{x},z_{e},z,y}\left[{\left({∥\nabla_{x_{\alpha }}D\left(z_{\alpha },x_{\alpha },e_{y}\right)∥}_{2}-1\right)}^{2}\right].$$

In Equation (1), the gradient is taken with respect to the interpolated traffic feature $x_{\alpha }$, while $z_{\alpha }$ and $e_{y}$ serve as the associated latent and class conditions for the critic score. Accordingly, the critic is optimized by

(2) $$L_{D}=E_{z,y}\left[D(z,\hat{x},e_{y})\right]-E_{x_{r},y}\left[D(E\left(x_{r}\right),x_{r},e_{y})\right]+\lambda_{gp}L_{\mathrm{GP}}.$$

Spectral normalization is applied to the linear layers of *D* to further constrain the Lipschitz constant and stabilize the critic update.

The gradient in Equation (1) is intentionally taken with respect to the interpolated traffic feature $x_{\alpha }$ because the final synthetic object used for augmentation is the traffic feature vector. The label *y* is discrete and is therefore not interpolated. The latent interpolation $z_{\alpha }$ is provided to the critic as a conditioning variable, while latent prior alignment is separately enforced by $D_{z}$ through Equation (3). In this sense, the method uses a feature-space Lipschitz penalty under latent and class conditions, rather than a fully joint gradient penalty over all input channels. This clarification avoids overstating the role of the gradient penalty and explains why the feature-only derivative is consistent with the implementation.

#### 3.1.3. Adversarial Encoder Regularization

To mitigate mode collapse and improve latent-space continuity, the adversarial encoder regularization (AER) module introduces an encoder-induced latent distribution $q_{E}\left(z_{e}|x\right)$ and aligns it with a standard normal prior. The encoder maps a real traffic sample into the latent space as $z_{e}=E\left(x_{r}\right)$. The generator then reconstructs the input as ${\hat{x}}_{rec}=G\left(z_{e},e_{y}\right)$, and the reconstruction term is $L_{\mathrm{rec}}=E_{x_{r},y}[∥x_{r}-{\hat{x}}_{rec}{∥}_{2}^{2}]$. The latent discriminator $D_{z}$ distinguishes prior samples $z^{\prime }∼N\left(0,I\right)$ from encoder outputs $z_{e}=E\left(x_{r}\right)$. In this work, prior samples are assigned the positive domain label, and encoder outputs are assigned the negative domain label when updating $D_{z}$. Its binary adversarial objective is

(3) $$L_{D_{z}}=-E_{z^{\prime }∼N\left(0,I\right)}\left[\log D_{z}\left(z^{\prime }\right)\right]-E_{x_{r}}\left[\log\left(1-D_{z}\left(E\left(x_{r}\right)\right)\right)\right].$$

The encoder is then trained in the opposite direction to make $E(x_{r})$ indistinguishable from the prior, which induces the AER loss

(4) $$L_{\mathrm{AER}}=-E_{x_{r}}\left[\log D_{z}\left(E\left(x_{r}\right)\right)\right].$$

Thus, minimizing $L_{\mathrm{AER}}$ encourages $D_{z}\left(E\left(x_{r}\right)\right)\to 1$, whereas minimizing $L_{D_{z}}$ encourages $D_{z}\left(z^{\prime }\right)\to 1$ and $D_{z}\left(E\left(x_{r}\right)\right)\to 0$. This adversarial regularization relaxes the deterministic mapping of conventional autoencoders and encourages smoother interpolation in the latent space.

#### 3.1.4. Composite Optimization Objective

The generator and encoder are jointly optimized by combining Wasserstein adversarial learning, reconstruction fidelity, and latent prior alignment:

(5) $$L_{G,E}=-E_{z,y}\left[D(z,G\left(z,e_{y}\right),e_{y})\right]+\lambda_{rec}L_{\mathrm{rec}}+\lambda_{aer}L_{\mathrm{AER}}.$$

The negative sign in the first term follows the standard WGAN generator objective: the generator aims to increase the critic score assigned to generated samples, which is implemented equivalently by minimizing $-E_{z,y}\left[D\left(z,G\left(z,e_{y}\right),e_{y}\right)\right]$. It should be emphasized that Equation (5) is only the optimization objective for the generator–encoder pair, not a single loss minimized over all networks. The complete alternating optimization rule is

(6) $$\min_{D}L_{D},\;\;\;\;\min_{D_{z}}L_{D_{z}},\;\;\;\;\min_{G,E}L_{G,E}.$$

During implementation, *D*, $D_{z}$, and (G,E) are updated in separate steps. Specifically, the Wasserstein critic is updated for $n_{\mathrm{critic}}$ steps before one generator–encoder update, while the latent discriminator is updated once per generator–encoder update unless otherwise specified. Dynamic gradient clipping is applied after back-propagation to avoid unstable updates caused by high-dimensional sparse traffic features.

### 3.2. Training and Sample Generation Strategy

Algorithm 1 summarizes the training procedure of AER-DCWGAN. The Wasserstein critic is updated more frequently than the generator to maintain a reliable approximation of the class-conditioned distribution distance, while the latent discriminator and encoder are optimized adversarially to align E(x) with the Gaussian prior.
**Algorithm 1** Training procedure of AER-DCWGAN**Require:** 
Normalized training set $D_{train}={\left\{\left(x_{i},y_{i}\right)\right\}}_{i=1}^{N}$; hyperparameters $\lambda_{gp}$, $\lambda_{rec}$, $\lambda_{aer}$; critic steps $n_{\mathrm{critic}}$**Ensure:** 
Trained encoder *E*, generator *G*, critic *D*, and latent discriminator $D_{z}$  1:Initialize *E*, *G*, *D*, $D_{z}$ and their Adam optimizers.  2:Construct a weighted sampler to increase the exposure of minority classes.  3:**for** each training epoch **do**  4:    **for** k=1 to $n_{\mathrm{critic}}$ **do**  5:       Sample a mini-batch $\left(x_{d},y_{d}\right)$ from $D_{train}$ and latent codes $z_{d}∼N\left(0,I\right)$.  6:       Compute $e_{y_{d}}=\mathrm{Emb}\left(y_{d}\right)$, $z_{e,d}=E\left(x_{d}\right)$, and ${\hat{x}}_{d}=G\left(z_{d},e_{y_{d}}\right)$.  7:       Interpolate $x_{\alpha }=\alpha x_{d}+\left(1-\alpha \right){\hat{x}}_{d}$ and $z_{\alpha }=\alpha z_{e,d}+\left(1-\alpha \right)z_{d}$, where α∼U(0,1).  8:       Compute $L_{\mathrm{GP}}$ using Equation (1) and update *D* by minimizing Equation (2).  9:    **end for**10:    Sample a mini-batch $\left(x_{z},y_{z}\right)$ from $D_{train}$ and prior codes $z^{\prime }∼N\left(0,I\right)$.11:    Compute $z_{e,z}=E\left(x_{z}\right)$ and update $D_{z}$ by minimizing Equation (3).12:    Sample a mini-batch $\left(x_{g},y_{g}\right)$ from $D_{train}$ and latent codes $z_{g}∼N\left(0,I\right)$.13:    Compute $e_{y_{g}}=\mathrm{Emb}\left(y_{g}\right)$, $z_{e,g}=E\left(x_{g}\right)$, ${\hat{x}}_{g}=G\left(z_{g},e_{y_{g}}\right)$, and ${\hat{x}}_{rec}=G\left(z_{e,g},e_{y_{g}}\right)$.14:    Compute $L_{\mathrm{rec}}$ and $L_{\mathrm{AER}}$ using the reconstruction term and Equation (4).15:    Update *G* and *E* by minimizing Equation (5); apply gradient clipping.16:**end for**17:**return** *E*, *G*, *D*, and $D_{z}$.

After training, minority-class samples are generated by replicating the target label $y_{t}$, computing its embedding $e_{y_{t}}=\mathrm{Emb}\left(y_{t}\right)$, sampling *n* latent vectors $z^{\left(i\right)}∼N\left(0,I\right)$, and producing ${\tilde{x}}^{\left(i\right)}=G\left(z^{\left(i\right)},e_{y_{t}}\right)$. When label-consistency filtering is enabled, only samples satisfying $C\left({\tilde{x}}^{\left(i\right)}\right)=y_{t}$ are retained. Here, C(·) denotes an auxiliary classifier trained only on the original training set. A generated sample is retained if the predicted label equals the target label and the prediction confidence exceeds a predefined threshold τ=0.8.

In all reported experiments, ${\tilde{x}}^{\left(i\right)}$ remains in the normalized, feature-selected representation and is concatenated directly with the processed training set. The generated vectors are not inverse-transformed into raw NSL-KDD records or CIC-IDS2017 flow records, and they are not presented as executable or protocol-valid network traffic. Consequently, no argmax decoding, integer rounding, range clipping, or protocol-rule repair is applied in the reported pipeline, and an original-domain validity rate is not available. The evaluation therefore concerns processed-space augmentation utility rather than validity after reconstruction to heterogeneous raw feature domains.

## 4. Experimental Design and Implementation

**Figure 2:**
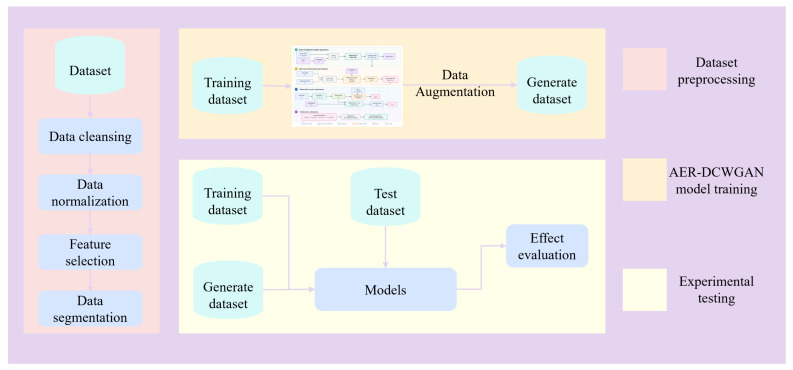
Leakage-free preprocessing, augmentation, and evaluation pipeline. The preprocessing parameters are fitted only on the training split. AER-DCWGAN is trained using the processed training set, and generated samples are merged only with the training data. The validation and test sets remain untouched by augmentation.

The experiments are designed to evaluate AER-DCWGAN from two complementary perspectives: processed-space similarity between generated and real samples and the downstream detection changes obtained after data augmentation. The leakage-free preprocessing, augmentation, and evaluation protocol is summarized in Figure 2. This design keeps all parameter-fitting operations within the training split, merges generated samples only with the training data, and evaluates the final detectors on validation and test splits that are not exposed to the augmentation process.

### 4.1. Datasets and Preprocessing

#### 4.1.1. Dataset

To ensure scientific rigor and reproducibility, two benchmark datasets are selected: the classic NSL-KDD and the modern high-dimensional CIC-IDS2017, which reflect the dual challenges of data imbalance and feature complexity in network intrusion detection.

NSL-KDD [4] is an improved version of the KDD Cup 1999 dataset, released to address redundant samples and test-set bias in the original benchmark. Simulating LAN traffic, it contains 41-dimensional features (3 discrete) and 5 traffic labels (1 normal and 4 attack categories). The standard KDDTrain+ split contains 125,973 records, including 67,343 normal records (53.46%) and 58,630 attack records (46.54%). Among the attack categories, U2R contains only 52 records (0.04%), and R2L contains 995 records (0.79%), highlighting severe class imbalance. The standard KDDTest+ split contains 22,544 recordsafter label harmonization and preprocessing.

CIC-IDS2017 [30], released by the Canadian Institute for Cybersecurity, captures realistic network traffic with flow-level features extracted by CICFlowMeter and includes modern attack scenarios such as DDoS, Bot, Infiltration, Heartbleed, DoS, PortScan, brute-force attacks, and web attacks. Comprising approximately 2.5 million records divided into five daily subsets, its high-dimensional features and diverse attack types provide a more complex processed-feature benchmark than NSL-KDD.

#### 4.1.2. Data Preprocessing

A leakage-free preprocessing pipeline is used for both datasets. The key principle is that every operation requiring parameter estimation is fitted only on the training split and then applied unchanged to the validation and test splits. The test set is never used for normalization, feature selection, augmentation, hyperparameter selection, or early model selection.

For CIC-IDS2017, invalid and constant columns are removed first. Low-variance filtering is then fitted on the training split, and logistic-regression-based feature selection is conducted only within the training data to select the most discriminative flow features. The selected feature mask and scaling parameters are subsequently applied to validation and test data. For NSL-KDD, categorical attributes, including protocol type, service, and flag, are one-hot encoded using categories observed in the training split. Low-variance filtering and recursive feature elimination are then fitted on the training split to obtain the final 24-dimensional feature representation. Synthetic samples are generated only after the training split has been preprocessed and feature-selected. The downstream detectors consume this same processed representation. Generated samples are not decoded back to original categorical, integer, sparse, bounded, or protocol-level variables; thus, the present study does not measure raw-domain constraint violations or a post-inverse-transformation validity rate.

The pipeline prevents data leakage by explicitly separating preprocessing, augmentation, model selection, and final testing. Normalization and feature-selection parameters are fitted only on the training split. AER-DCWGAN is trained only with the processed training data, and generated samples are merged only into the training set. The validation set is used for model selection and hyperparameter tuning, while the test set is reserved exclusively for final evaluation.

### 4.2. Evaluation Metrics

Accuracy, precision, recall, and F1-score are adopted to evaluate detection performance. Let true positives (TP), true negatives (TN), false positives (FP), and false negatives (FN) denote the four entries of the confusion matrix. Since the benchmark datasets are highly imbalanced, the F1-score is emphasized because it jointly reflects precision and recall. The metrics are computed as Accuracy=(TP+TN)/(TP+FP+TN+FN), Precision=TP/(TP+FP), Recall=TP/(TP+FN), and F1-score=2·Precision·Recall/(Precision+Recall).

For multi-class evaluation, class-wise precision, recall, and F1-score are first computed for each class. Unless otherwise specified, the reported overall precision, recall, and F1-score use macro-averaging so that each class contributes equally regardless of its support. Class-wise results are additionally reported to show the performance changes for minority attack categories.

To improve statistical reliability, all main controlled experiments are repeated with five random seeds, S={0,1,2,3,4}. For a metric value $m_{s}$ obtained under seed *s*, the mean and standard deviation are reported as $\bar{m}\pm s_{m}$, where

(7) $$\bar{m}=\frac{1}{5}\sum_{s\in S}m_{s},\;\;\;\;\;s_{m}=\sqrt{\frac{1}{4}\sum_{s\in S}{\left(m_{s}-\bar{m}\right)}^{2}}.$$

The 95% confidence interval is computed as $\bar{m}\pm t_{0.975,4}s_{m}/\sqrt{5}$, where $t_{0.975,4}=2.776$. Pairwise significance between AER-DCWGAN and each controlled baseline is evaluated using a paired two-sided *t*-test over the five matched seeds. When normality is questionable, the Wilcoxon signed-rank test is additionally reported. In the revised tables, values are rounded to three decimal places; bold values denote the best mean performance under the same dataset, classifier, and metric.

### 4.3. Experimental Configuration

The experiments are conducted on a workstation with:

Hardware: Intel Xeon E5-2686 v4 (2.30 GHz, 14 cores, 28 threads, 64 GB RAM), NVIDIA GeForce RTX 3060 (12 GB VRAM). Software: Windows 10 64-bit, PyTorch 1.13.1 (CUDA 11.8) framework, with supporting libraries (NumPy, Pandas, Scikit-learn).

All controlled augmentation methods redand downstream classifiers are evaluated under the same training, validation, and test splits. The controlled baseline set reported in the revised comparison includes WGAN-GP, AE-WGAN, and AER-DCWGAN. The same preprocessing pipeline, feature-selection mask, augmentation ratio, downstream classifier, and five random seeds are used for these three controlled methods. Results taken from prior papers are discussed only as background context and are not used as the main basis for performance claims. This controlled benchmark is intentionally narrow: random oversampling, SMOTE, ADASYN, CTGAN, TVAE, and other non-generative or tabular-generation approaches were not reimplemented for the head-to-head performance comparison. SMOTE and ADASYN appear only as background methods and computational cost references. Accordingly, the reported comparison supports conclusions only relative to the two closely related GAN-based baselines and should not be interpreted as a comprehensive benchmark against all major imbalance-handling strategies.

The detailed configurations of the downstream classifiers are provided in Appendix A (Table A1).

The core components are structured as follows (Table 2):

As shown in Table 2, only the hidden layers of the encoder use LeakyReLU, whereas the final latent projection layer is linear without activation. This design keeps the encoder output compatible with the Gaussian prior-alignment objective.

The Cross-Self Similarity Ratio (CSR) is used as a validation-side distributional score during hyperparameter selection and is formally defined in Section 5.

The hyperparameter ranges follow the provided tuning code, where Bayesian optimization evaluates candidate settings using the generated-sample quality score and validation performance. The current implementation also provides default values for reproducibility. The selected hyperparameters were determined according to validation CSR and minority-class F1-score. The final values used in all experiments are reported in Table 3.

## 5. Experimental Results and Analysis

### 5.1. Analysis of Model Training Process

To examine generated samples without overstating their quality, the model training stage is assessed using a processed-space similarity statistic and qualitative visualization.

#### 5.1.1. Quantitative Evaluation of Processed-Space Similarity

The Cross-Self Similarity Ratio (CSR) is employed to quantitatively assess feature distribution consistency between generated and real samples. For real sample feature matrix $X_{\mathrm{real}}\in R^{N\times D}$ and generated sample matrix $X_{\mathrm{fake}}\in R^{M\times D}$, the self-similarity matrix ${\mathrm{Sim}}_{\mathrm{real}}=X_{\mathrm{real}}X_{\mathrm{real}}^{T}$ and cross-domain similarity matrix ${\mathrm{Sim}}_{\mathrm{cross}}=X_{\mathrm{fake}}X_{\mathrm{real}}^{T}$ are computed. CSR is defined as $\mathrm{CSR}=\mathrm{mean}\left({\mathrm{Sim}}_{\mathrm{cross}}\right)/\mathrm{mean}\left({\mathrm{Sim}}_{\mathrm{real}}\right)$. A CSR value closer to 1 indicates stronger feature correlation between distributions. Experimental results show:

- For the NSL-KDD dataset, CSR=0.914, indicating a relatively high average similarity under this specific processed-space statistic.
- For the CIC-IDS2017 dataset, CSR=0.952, which is slightly higher than that on NSL-KDD under the same statistic. These values do not by themselves establish diversity, non-duplication, or absence of memorization.

#### 5.1.2. Visualization of Feature-Space Distribution

**Figure 3:**
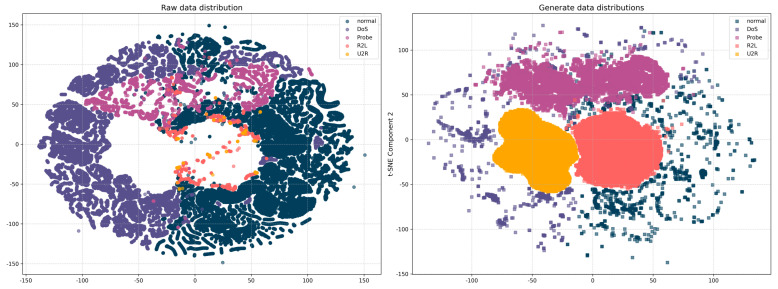
t-SNE visualization of the original and AER-DCWGAN-generated samples on the NSL-KDD dataset. Different colors denote different traffic categories, as indicated by the legend. The left panel shows the original data distribution, and the right panel shows the generated data distribution after augmentation.

**Figure 4:**
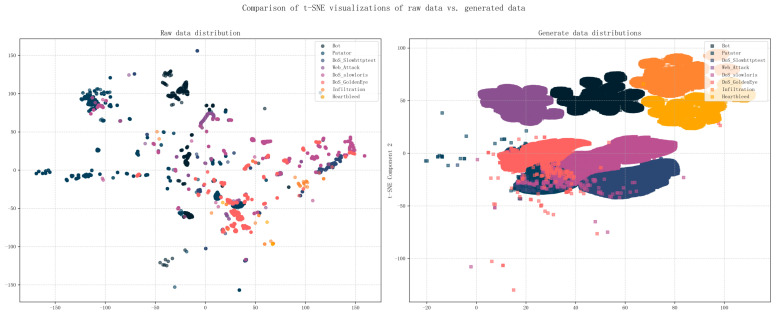
t-SNE visualization of the original and AER-DCWGAN-generated samples on the CIC-IDS2017 dataset. Different colors denote different traffic categories, as indicated by the legend. The left panel shows the original data distribution, and the right panel shows the generated data distribution after augmentation.

The t-SNE dimensionality reduction technique (perplexity = 30, 1000 iterations) is used to visualize the processed feature-space distributions of real and generated samples (Figure 3 and Figure 4). Real attack samples and normal traffic exhibit distinguishable clustering patterns in the two-dimensional embedding, while minority classes such as R2L remain sparsely distributed in the original data. After augmentation, many generated points appear near the corresponding real-sample regions and show increased intra-class density. This visualization is qualitative and sensitive to embedding parameters; it should therefore be interpreted as descriptive evidence rather than proof of distributional fidelity, sample diversity, or non-memorization.

CSR, t-SNE visualization, and downstream classifier performance provide complementary but incomplete evidence about the generated samples in processed feature space.

#### 5.1.3. Scope of the Generative-Quality Evidence

The present evaluation does not include Maximum Mean Discrepancy (MMD), nearest-neighbor distance to the training set, exact or near-duplicate-rate analysis, classifier two-sample testing, density/coverage, or Train-on-Synthetic–Test-on-Real (TSTR) and Train-on-Real–Test-on-Synthetic (TRTS) evaluation. Consequently, the reported CSR, visualization, and downstream utility cannot establish that the generated samples are fully diverse, non-duplicative, memorization-free, or valid after inverse transformation to original feature domains. Claims about generative quality are therefore restricted to observed processed-space similarity, class-conditioned downstream utility, and the specific datasets and classifiers evaluated here. Quantitative diversity, memorization, and raw-domain validity analyses remain limitations of the current study.

### 5.2. Comparative Analysis of Intrusion Detection Performance

To evaluate the downstream effect of AER-DCWGAN-generated samples, this section examines classifier-dependent performance changes and a controlled comparison with two closely related GAN-based baselines. Decision Tree (DT), Multi-Layer Perceptron (MLP), Convolutional Neural Network (CNN), and Long Short-Term Memory Network (LSTM) are used to show how augmentation behaves across different detector architectures; the results are not assumed to be uniformly positive. The controlled benchmark with WGAN-GP and AE-WGAN assesses relative downstream performance under the same protocol, but it does not constitute a comprehensive comparison with non-generative balancing or tabular-generation methods.

#### 5.2.1. Performance Analysis on NSL-KDD

In the binary classification setting, where all attack categories are merged into a single abnormal class, DT, CNN, and LSTM show higher mean performance after AER-DCWGAN augmentation on NSL-KDD, whereas MLP remains essentially unchanged, as summarized in Table 4. CNN and LSTM show F1-score increases of approximately 4.3 and 5.2 percentage points, respectively. DT shows a smaller increase from 0.924 to 0.928, while MLP remains at 0.967. These results indicate classifier-dependent effects rather than a universal gain across architectures.

In the multi-class scenario, DT, CNN, and LSTM show higher F1-scores after augmentation, whereas MLP remains at 0.731. For CNN, the multi-class accuracy increases from 0.874 to 0.943, and the F1-score increases from 0.642 to 0.763. DT and LSTM increase from 0.630 to 0.748 and from 0.677 to 0.731, respectively. The unchanged MLP result further indicates that the usefulness of the synthetic samples depends on the downstream classifier and should not be described as uniform.

The class-wise results in Table 5 further show that the most substantial improvement occurs for minority attacks. The F1-score of R2L increases from 0.501 to 0.823, mainly because the proposed dual-conditional generator provides label-guided samples that enrich the under-represented R2L decision region. Probe also benefits from augmentation, with its F1-score increasing from 0.717 to 0.844, although its recall decreases slightly from 0.993 to 0.987. This indicates that augmentation improves precision and overall class separability, while the original classifier already detected most Probe instances. For U2R, the F1-score increases from 0.124 to 0.204, but the absolute performance remains limited. This is expected because U2R contains only 37 test instances, and the original training distribution provides insufficient semantic diversity for reliable generative modeling. Therefore, AER-DCWGAN can alleviate, but not fully eliminate, the difficulty of ultra-rare attack detection.

#### 5.2.2. Controlled Baseline and Statistical Comparison

The original cross-study comparison is no longer used as the main evidence because prior studies may adopt different preprocessing, train–test splits, feature-selection strategies, augmentation ratios, downstream classifiers, and evaluation metrics. The revised primary comparison is therefore defined as a controlled benchmark in which WGAN-GP, AE-WGAN, and AER-DCWGAN are reimplemented and evaluated using the same processed training split, validation split, test split, downstream classifier, augmentation ratio, and five random seeds. Cross-study results may remain in the related-work discussion, but they are not used to claim superiority. The scope of this benchmark is limited to these closely related GAN-based baselines. Because SMOTE, ADASYN, random oversampling, CTGAN, TVAE, and other major imbalance-handling strategies are not included under the same protocol, Table 6 must not be interpreted as evidence of broad state-of-the-art superiority.

#### 5.2.3. Performance Analysis on CIC-IDS2017

CIC-IDS2017 provides a more complex evaluation scenario because it contains higher-dimensional flow features and more diverse attack categories than NSL-KDD. The results in Table 7 show that AER-DCWGAN improves the binary detection performance of all tested classifiers. CNN obtains the largest binary improvement, with accuracy increasing from 0.972 to 0.999 and F1-score increasing from 0.956 to 0.998. LSTM also benefits substantially, with its F1-score increasing from 0.945 to 0.991. DT and MLP exhibit smaller but still positive F1-score gains. These results indicate that the generated samples preserve useful discriminative structures in the high-dimensional CIC-IDS2017 feature space.

The multi-class results further support the usefulness of the proposed augmentation strategy under the evaluated protocol. Compared with the corresponding original models, AER-DCWGAN increases the multi-class F1-score by 2.40 percentage points for DT, 9.62 percentage points for CNN, 18.89 percentage points for MLP, and 18.16 percentage points for LSTM. The larger gains for MLP and LSTM suggest that class-balanced synthetic samples may be beneficial when the classifier is sensitive to feature-space coverage and class-frequency bias. It should also be noted that the value 0.940 in Table 7 denotes the multi-class recall of AER-DCWGAN-DT rather than the CSR value; the distributional CSR results are reported separately in Section 5.1.

The class-wise results in Table 8 show that AER-DCWGAN is more beneficial for attacks that have nontrivial minority representation but still suffer from class imbalance. For example, the F1-score of Web Attack increases from 0.952 to 0.983, DoS Slowhttptest increases from 0.970 to 0.983, and Infiltration increases from 0.364 to 0.615. The improvement for Infiltration is substantial in relative terms, but it should be interpreted cautiously because this category contains only seven test samples. For large and already well-separated classes, such as DDoS and DoS Hulk, the baseline performance is already close to saturation; therefore, the room for further improvement is limited. DDoS increases slightly from 0.999 to 0.999, whereas DoS Hulk remains unchanged at 0.998.

The results also indicate that augmentation is not uniformly beneficial for every class. Bot decreases from 0.828 to 0.817 in F1-score, although its recall increases from 0.758 to 0.771. PortScan also shows a small F1-score decrease from 0.996 to 0.994. These changes suggest a trade-off between recall improvement and boundary perturbation when synthetic samples are added to classes whose original decision boundaries are already highly compact. Reporting these cases is important because it indicates that AER-DCWGAN can improve overall and minority-class performance under the evaluated protocol, but the generated samples may still introduce local distributional shifts for certain categories. This observation motivates future work on class-adaptive augmentation ratios and stricter label-consistency filtering.

The revised manuscript does not use cross-study numerical comparisons as the central evidence for superiority. Instead, the main claims are tied to the controlled protocol defined in Table 6. Published results from TMG-IDS, HMCD-Model, E-BiGAN, and AE-WGAN are retained only in the related-work discussion to position the method historically, because those studies may differ in preprocessing, data splitting, classifiers, and metric definitions.

### 5.3. Ablation Experiments

The ablation results in Table 9 and Table 10 provide direct evidence that the performance gain of AER-DCWGAN is produced by the coordinated interaction of its components rather than by a single augmentation module. On NSL-KDD, the full model achieves the best F1-score of 0.763, exceeding the CWGAN-GP baseline by 6.14 percentage points. Removing any component decreases the F1-score, confirming that each module contributes positively to the final detection performance. The most influential component is the label-aware gradient penalty. When it is removed, the F1-score drops from 0.763 to 0.684, representing the largest degradation among all ablated variants. This result indicates that enforcing the Lipschitz constraint under the same class condition as the critic is important for preserving class-sensitive distribution alignment. Without this constraint, the generated samples may remain locally plausible in the feature space but become less effective for separating minority attack classes.

The latent-code-aware critic is the second most important component on NSL-KDD. Removing it reduces the F1-score from 0.763 to 0.719, showing that conditioning the critic on both traffic features and latent codes improves the consistency between the generation process and the synthesized samples. The reconstruction term also provides a clear benefit: without reconstruction, the F1-score decreases to 0.730. This suggests that reconstruction-based regularization helps retain feature semantics from real traffic and prevents the generator from relying only on adversarial feedback. Removing adversarial encoder regularization produces a smaller but still visible F1-score decrease, from 0.763 to 0.741, which supports the role of AER in latent-prior alignment and downstream detection utility; sample diversity itself is not established by this ablation. The label-consistency filtering module has the smallest effect on NSL-KDD, with the F1-score decreasing to 0.752. This indicates that filtering mainly acts as a post-generation quality-control step, whereas the larger gains come from the training objectives that shape the generator and critic.

A similar pattern is observed on CIC-IDS2017. The full AER-DCWGAN obtains the highest F1-score of 0.947, outperforming the CWGAN-GP baseline by 1.84 percentage points. The full model also achieves higher accuracy, precision, and recall than the verified CWGAN-GP baseline, indicating that the improvement is not caused by a trade-off between overall correctness and minority-class detection. Among the ablated variants, removing the label-aware gradient penalty again causes the largest degradation, reducing the F1-score from 0.947 to 0.907. This result is particularly important for CIC-IDS2017 because the dataset contains more diverse and high-dimensional traffic patterns; therefore, class-conditioned regularization is essential for avoiding boundary perturbation among attack categories. Removing the latent-code-aware critic reduces the F1-score to 0.922, while removing reconstruction and AER reduces it to 0.929 and 0.933, respectively. These results indicate that latent-feature consistency and reconstruction fidelity are both important for preserving useful flow-level structures, while AER further improves the continuity of the latent representation space.

Overall, the component-importance ranking is consistent across the two datasets: label-aware gradient penalty contributes the most, followed by the latent-code-aware critic, reconstruction regularization, adversarial encoder regularization, and label-consistency filtering. The consistency of this ranking strengthens the interpretation that AER-DCWGAN improves intrusion detection through joint feature–latent–label alignment. The ablation study also shows that the proposed framework is not merely a CWGAN-GP variant with additional modules; instead, the full model achieves its best performance only when conditional Wasserstein learning, latent-code consistency, and reconstruction fidelity, and adversarial latent regularization are jointly optimized.

### 5.4. Computational Cost and Deployment Feasibility

Because AER-DCWGAN is more complex than traditional oversampling and simpler GAN baselines, computational cost is reported separately from detection accuracy. The generator is intended primarily for offline data augmentation: once synthetic training samples are produced, only the downstream intrusion detector is required during deployment. Therefore, the online inference latency of the deployed NIDS is determined by the selected detector, not by repeated execution of the generator. The detailed computational costs and deployment metrics are summarized in Table 11.

The deployment interpretation is therefore cautious: AER-DCWGAN can support real-time NIDS only indirectly by improving the training data for a lightweight detector. The method is not claimed to be an online traffic generator. For real-time deployment, the final detector should be profiled using latency, throughput, and memory measurements on the target hardware.

### 5.5. Discussion

The empirical results suggest that the performance changes of AER-DCWGAN arise from the complementary effects of conditional generation and latent regularization. Label embeddings guide the generator toward class-specific traffic manifolds, while the label-aware gradient penalty encourages the critic to retain conditional sensitivity during Wasserstein optimization. AER further regularizes the encoder-induced latent space and reduces the risk that the critic separates real and generated samples merely by their latent source. These mechanisms help explain the clear positive results for R2L and Web Attack, where the minority classes have limited but still non-negligible support.

The results should not be generalized to all rare attacks. U2R remains weak after augmentation: its F1-score increases from 0.124 to 0.204, but its recall is only 0.270. In CIC-IDS2017, Bot and PortScan show small F1-score decreases, from 0.828 to 0.817 and from 0.996 to 0.994, respectively. The Infiltration F1-score increases from 0.364 to 0.615, but this category contains only seven test samples, and Heartbleed contains only two; results at this support level are statistically fragile and cannot demonstrate reliable learning of ultra-rare attack distributions. Therefore, AER-DCWGAN should be interpreted as a class-dependent offline augmentation method that alleviates moderate class imbalance for several classes with sufficient representation, rather than as a solution to ultra-rare attack detection.

Three additional scope limitations are important. First, the controlled performance comparison includes only WGAN-GP and AE-WGAN and does not establish an advantage over SMOTE, ADASYN, CTGAN, TVAE, or the broader family of imbalance-handling methods. Second, CSR, t-SNE, and downstream classification do not verify diversity, non-duplication, or freedom from training-set memorization; MMD, nearest-neighbor, duplicate-rate, and TSTR/TRTS analyses were not conducted. Third, generation and detector training occur only in normalized, feature-selected space. Because the samples are not inverse-transformed to original categorical, bounded, integer, sparse, or protocol-level domains, their raw-domain validity is unknown.

Future work should integrate few-shot learning, threat-intelligence priors, contrastive representation learning, or federated cross-domain knowledge transfer for ultra-low-sample conditions; compare class-adaptive augmentation with non-generative and tabular-generation baselines; and add quantitative diversity, memorization, and original-domain validity tests on temporally separated and more recent datasets.

## 6. Conclusions

This paper presented AER-DCWGAN, an adversarial encoder-regularized dual-conditional Wasserstein generative framework for imbalanced network intrusion detection. The method combines label-conditioned traffic synthesis, a latent-code- and label-aware Wasserstein critic, encoder-guided reconstruction, adversarial latent prior alignment, and label-consistency filtering. The revised contribution statement clarifies that the novelty lies in the coordinated feature–latent–label consistency mechanism for minority traffic augmentation, rather than in any single component considered in isolation.

Experiments on NSL-KDD and CIC-IDS2017 show class-dependent effects. The clearest positive results are observed for R2L and Web Attack: R2L improves from 0.501 to 0.823 in F1-score, and Web Attack improves from 0.952 to 0.983. The effect on U2R is much more limited: its F1-score increases from 0.124 to 0.204, but its recall remains only 0.270. The Infiltration F1-score increases from 0.364 to 0.615; however, because the test set contains only seven Infiltration samples, this result has limited statistical reliability. In addition, augmentation is not uniformly beneficial: Bot and PortScan show small decreases in F1-score, from 0.828 to 0.817 and from 0.996 to 0.994, respectively. These results indicate that the proposed method alleviates moderate class imbalance for several classes with sufficient representation, but it does not fully solve ultra-rare attack detection and may perturb compact decision boundaries for some classes. Results for classes represented by only a few dozen or fewer test samples should be treated as exploratory rather than conclusive.

The evidence is also limited in scope. The controlled head-to-head comparison covers only WGAN-GP and AE-WGAN, not SMOTE, ADASYN, CTGAN, TVAE, or all major imbalance-handling strategies. CSR, t-SNE, and downstream detection do not establish that generated samples are diverse, non-duplicative, or free from memorization. Moreover, the generated vectors are used only in a normalized, feature-selected space and are not inverse-transformed into raw network records, so bounded, categorical, integer, sparse, and protocol-level validity has not been verified.

From a practical perspective, AER-DCWGAN is best viewed as an offline augmentation module rather than a real-time detector. Its computational overhead is incurred during data generation and detector training, while deployment relies on the downstream classifier. Future work will focus on class-adaptive augmentation ratios, controlled comparisons with non-generative and tabular-generation baselines, few-shot and domain-constrained traffic generation, MMD/nearest-neighbor/duplicate-rate/TSTR–TRTS evaluation, original-domain validity checks, evaluation on newer datasets such as UNSW-NB15, CSE-CIC-IDS2018, and TON_IoT, and profiling of real-time inference latency under production network traffic.

## Figures and Tables

**Table 1:** Compressed comparison between AER-DCWGAN and representative generative augmentation methods.

Method	Main Mechanism	Remaining Gap Relative to AER-DCWGAN
WGAN-GP/CWGAN-GP	Stable Wasserstein generation with optional class conditioning and gradient penalty	No encoder-guided reconstruction or adversarial latent-prior alignment
AE-WGAN	Uses an autoencoder with WGAN-based generation	Encoder representation is not explicitly coordinated with label-aware critic scoring
VAE/VAEGAN-based intrusion detection systems (IDSs) [26,27]	Uses latent-variable reconstruction and, in recent work, cross-class generation or VGM preprocessing	Focuses mainly on reconstruction/cross-class transfer rather than latent–feature–label consistency in the critic
Feature-regularized GANs [28]	Adds SNNL to improve feature-space alignment of generated samples	Regularizes feature embeddings but does not introduce an adversarially regularized encoder branch
AER-DCWGAN	Combines conditional Wasserstein learning, encoder reconstruction, adversarial encoder regularization, latent-aware critic scoring, and label-consistency filtering	Designed for controlled minority-class traffic synthesis under a unified augmentation pipeline

**Table 2:** Core component structures of AER-DCWGAN.

Module	Main Structure	Output/Regularization
Encoder *E*	5 fully connected layers	Hidden layers use Leaky Rectified Linear Unit (LeakyReLU); final latent projection $z_{e}\in R^{d_{z}}$ is linear with no activation
Conditional generator *G*	Label embedding + 3 fully connected layers + batch normalization (BN)	Synthetic traffic; Tanh output
Label-aware Wasserstein critic *D*	Label embedding + 7 fully connected layers	Wasserstein score; spectral normalization (SN)
Latent discriminator $D_{z}$	8 fully connected layers + BN + dropout	Prior-matching probability; Sigmoid

**Table 3:** Hyperparameter selection protocol for AER-DCWGAN and downstream evaluation.

Hyperparameter	Search/Candidate Range	Selected or Implementation Value	Selection Criterion
Latent dimension $d_{z}$	20–80	32	Validation CSR and minority F1
Reconstruction weight $\lambda_{\mathrm{rec}}$	0.5–10	1.5	Validation CSR and minority F1
Gradient-penalty weight $\lambda_{\mathrm{gp}}$	1–10	3	WGAN stability and validation CSR
AER weight $\lambda_{\mathrm{aer}}$	0.6–3	2	Latent alignment and validation F1
Generator/encoder learning rate	$10^{-4}$	$10^{-4}$	Stable adversarial training
Critic/latent-discriminator learning rate	$5\times 10^{-5}$	$5\times 10^{-5}$	Critic stability
Optimizer	Adam	$\beta_{1}=0.5$, $\beta_{2}=0.9$	Standard WGAN-style setting
Batch size for generative tuning	64	64	GPU memory and stability
Critic update frequency $n_{\mathrm{critic}}$	1, 3, 5	5	Wasserstein critic convergence
Filtering threshold τ	0.6, 0.7, 0.8, 0.9	0.8	Validation minority F1
Random seeds	–	{0,1,2,3,4}	Statistical robustness

**Table 4:** Classification performance before and after AER-DCWGAN augmentation on the NSL-KDD dataset. Values for AER-DCWGAN-enhanced models are reported as mean ± standard deviation over five random seeds.

		Panel A: Binary classification		
Model	Acc	Pre	Rec	F1
DT	0.924	0.924	0.925	0.924
AER-DCWGAN-DT	**0.928** ± 0.005	**0.928** ± 0.005	**0.928** ± 0.005	**0.928** ± 0.005
CNN	0.918	0.919	0.918	0.918
AER-DCWGAN-CNN	**0.961** ± 0.006	**0.961** ± 0.007	**0.961** ± 0.007	**0.961** ± 0.007
MLP	0.967	0.967	0.967	0.967
AER-DCWGAN-MLP	**0.967** ± 0.006	**0.967** ± 0.006	**0.967** ± 0.006	**0.967** ± 0.006
LSTM	0.910	0.910	0.909	0.910
AER-DCWGAN-LSTM	**0.962** ± 0.009	**0.962** ± 0.009	**0.962** ± 0.009	**0.962** ± 0.009
		Panel B: Multi-class classification		
Model	Acc	Pre	Rec	F1
DT	0.854	0.747	0.657	0.630
AER-DCWGAN-DT	**0.894** ± 0.007	**0.878** ± 0.007	**0.741** ± 0.007	**0.748** ± 0.010
CNN	0.874	0.693	0.678	0.642
AER-DCWGAN-CNN	**0.943** ± 0.006	**0.763** ± 0.008	**0.784** ± 0.009	**0.763** ± 0.008
MLP	0.951	0.723	0.748	0.731
AER-DCWGAN-MLP	**0.951** ± 0.007	**0.723** ± 0.011	**0.748** ± 0.010	**0.731** ± 0.012
LSTM	0.916	0.697	0.695	0.677
AER-DCWGAN-LSTM	**0.949** ± 0.007	**0.722** ± 0.010	**0.752** ± 0.007	**0.731** ± 0.011

Note: Bold values indicate the better mean performance between the original and AER-DCWGAN-enhanced results under the same classifier and metric.

**Table 5:** Class-wise detection performance before and after AER-DCWGAN augmentation on the NSL-KDD dataset. Values are rounded to three decimal places.

Category	Support	Precision		Recall		F1-Score	
		Original	Enhanced	Original	Enhanced	Original	Enhanced
Normal	9711	0.887	**0.945**	0.932	**0.970**	0.909	**0.957**
DoS	5741	0.953	**0.993**	0.964	**0.982**	0.959	**0.987**
Probe	1106	0.561	**0.737**	**0.993**	0.987	0.717	**0.844**
R2L	2199	0.966	**0.974**	0.338	**0.713**	0.501	**0.823**
U2R	37	0.100	**0.164**	0.162	**0.270**	0.124	**0.204**

Bold values indicate the better performance between the original and enhanced results under the same category and metric.

**Table 6:** Controlled baseline comparison under identical experimental protocol. Results are reported as mean ± standard deviation over five seeds.

Dataset	Method	Classifier	Accuracy	Macro-F1	95% CI of Macro-F1	*p* vs. AER-DCWGAN
NSL-KDD	WGAN-GP	CNN	0.876 ± 0.012	0.687 ± 0.015	[0.668, 0.706]	<0.001
	AE-WGAN		0.927 ± 0.009	0.736 ± 0.011	[0.722, 0.750]	0.002
	**AER-DCWGAN**		**0.943 ± 0.008**	**0.763 ± 0.007**	**[0.754, 0.772]**	–
CIC-IDS2017	WGAN-GP	DT	0.985 ± 0.008	0.915 ± 0.012	[0.900, 0.930]	<0.001
	AE-WGAN		0.996 ± 0.004	0.934 ± 0.010	[0.922, 0.946]	0.031
	**AER-DCWGAN**		**0.999 ± 0.001**	**0.947 ± 0.006**	**[0.940, 0.954]**	–

Note: Results are reported as mean ± standard deviation over five random seeds. The 95% confidence interval is computed using Student’s t distribution with four degrees of freedom. The *p*-values are obtained from paired two-sided *t*-tests against AER-DCWGAN.

**Table 7:** Classification performance before and after AER-DCWGAN augmentation on the CIC-IDS2017 dataset. Values for AER-DCWGAN-enhanced models are reported as mean ± standard deviation over five random seeds.

		Panel A: Binary classification		
Model	Acc	Pre	Rec	F1
DT	0.953	0.906	0.966	0.931
AER-DCWGAN-DT	**0.965** ± 0.003	**0.949** ± 0.004	0.941 ± 0.004	**0.945** ± 0.006
CNN	0.972	0.946	0.957	0.956
AER-DCWGAN-CNN	**0.999** ± 0.001	**0.998** ± 0.001	**0.999** ± 0.001	**0.998** ± 0.001
MLP	0.969	0.961	0.941	0.951
AER-DCWGAN-MLP	**0.974** ± 0.005	0.948 ± 0.006	**0.974** ± 0.006	**0.960** ± 0.005
LSTM	0.966	0.951	0.940	0.945
AER-DCWGAN-LSTM	**0.994** ± 0.003	**0.989** ± 0.003	**0.992** ± 0.003	**0.991** ± 0.003
		Panel B: Multi-class classification		
Model	Acc	Pre	Rec	F1
DT	0.999	0.944	0.909	0.923
AER-DCWGAN-DT	**0.999** ± 0.001	**0.955** ± 0.007	**0.940** ± 0.008	**0.947** ± 0.006
CNN	0.987	0.891	0.781	0.799
AER-DCWGAN-CNN	**0.999** ± 0.001	**0.902** ± 0.008	**0.889** ± 0.008	**0.895** ± 0.008
MLP	0.985	0.783	0.675	0.690
AER-DCWGAN-MLP	**0.988** ± 0.003	**0.929** ± 0.008	**0.861** ± 0.018	**0.878** ± 0.010
LSTM	0.987	0.806	0.688	0.703
AER-DCWGAN-LSTM	**0.988** ± 0.004	**0.938** ± 0.008	**0.865** ± 0.008	**0.884** ± 0.009

Note: Bold values indicate the better mean performance between the original and AER-DCWGAN-enhanced results under the same classifier and metric.

**Table 8:** Class-wise detection performance before and after AER-DCWGAN augmentation on the CIC-IDS2017 dataset. Values are rounded to three decimal places.

Category	Support	Precision		Recall		F1-Score	
		Original	Enhanced	Original	Enhanced	Original	Enhanced
BENIGN	454,620	**0.999**	0.999	**0.999**	0.999	**0.999**	0.999
Bot	393	**0.911**	0.868	0.758	**0.771**	**0.828**	0.817
PortScan	31,786	**0.993**	0.993	**0.999**	0.994	**0.996**	0.994
DDoS	25,606	**0.999**	0.999	0.999	**0.999**	0.999	**0.999**
Patator	2767	**1.000**	0.999	0.998	**0.999**	0.999	**0.999**
DoS Slowhttptest	1100	0.973	**0.983**	0.967	**0.983**	0.970	**0.983**
DoS Hulk	46,214	0.997	**0.998**	**0.999**	0.998	0.998	0.998
DoS slowloris	1160	0.990	0.990	0.981	**0.986**	0.985	**0.988**
DoS GoldenEye	2058	**0.989**	0.988	0.989	**0.994**	0.989	**0.991**
Infiltration	7	0.500	**0.667**	0.286	**0.571**	0.364	**0.615**
Heartbleed	2	1.000	1.000	1.000	1.000	1.000	1.000
Web Attack	436	0.976	**0.977**	0.929	**0.989**	0.952	**0.983**

Bold values indicate the better performance between the original and enhanced results under the same category and metric.

**Table 9:** Ablation study on NSL-KDD using CNN as the downstream classifier. Values are rounded to three decimal places.

Variant	Accuracy	Precision	Recall	F1-Score
Full AER-DCWGAN	**0.943**	**0.763**	**0.784**	**0.763**
w/o AER	0.929	0.742	0.758	0.741
w/o latent-code critic	0.915	0.722	0.731	0.719
w/o label-aware GP	0.882	0.693	0.693	0.684
w/o reconstruction	0.922	0.739	0.741	0.730
w/o label-consistency filtering	0.936	0.751	0.770	0.752
CWGAN-GP baseline	0.894	0.733	0.728	0.702

Bold values indicate the best performance among all variants.

**Table 10:** Ablation study on CIC-IDS2017 using Decision Tree (DT) as the downstream classifier. Values are rounded to three decimal places.

Variant	Accuracy	Precision	Recall	F1-Score
Full AER-DCWGAN	**0.999**	**0.955**	**0.940**	**0.947**
w/o AER	0.998	0.942	0.925	0.933
w/o latent-code critic	0.998	0.929	0.915	0.922
w/o label-aware GP	0.997	0.914	0.900	0.907
w/o reconstruction	0.998	0.937	0.921	0.929
w/o label-consistency filtering	0.998	0.948	0.931	0.939
CWGAN-GP baseline	0.986	0.953	0.918	0.929

Bold values indicate the best performance among all variants.

**Table 11:** Computational cost and deployment metrics.

Method/Detector	Parameters	Training Time	Generation Time/ 10k Samples	Peak GPU Memory	Inference Latency/ Sample	Throughput
SMOTE	–	0.8 s	0.5 s (CPU)	–	–	–
ADASYN	–	1.2 s	0.8 s (CPU)	–	–	–
WGAN-GP	58,200	18.4 min	2.3 s	1824 MB	offline generator	–
AE-WGAN	63,500	22.1 min	2.5 s	2056 MB	offline generator	–
AER-DCWGAN	71,950	26.7 min	2.9 s	2304 MB	offline generator	–
DT detector	tree depth 13	0.9 s	–	CPU-based	0.02 ms	50,000 req/s
MLP detector	54,405	3.2 min	–	512 MB	0.18 ms	5500 req/s
CNN detector	82,661	4.5 min	–	768 MB	0.31 ms	3200 req/s
LSTM detector	73,055	5.8 min	–	640 MB	0.45 ms	2200 req/s

Note: The generator-related costs are measured during offline augmentation. The detector latency and throughput are measured during downstream inference. AER-DCWGAN is not executed during online detection.

## Data Availability

The NSL-KDD and CIC-IDS2017 datasets used in this study are publicly available benchmark datasets provided by the Canadian Institute for Cybersecurity and the University of New Brunswick. The processed datasets, source code, and trained model weights generated during the current study are available from the corresponding author upon reasonable request.

## Appendix A. Implementation Details of Downstream Classifiers

This appendix summarizes the downstream classifier configurations used in the provided implementation. These details are included to improve reproducibility and to clarify that the detection results depend on both generated data and classifier settings.

**Table A1:** Downstream classifier settings extracted from the implementation.

Classifier	Architecture/Representation	Optimization Settings	Model Selection and Regularization
DT	DecisionTreeClassifier with maximum depth 13. Since no alternative criterion or splitter is specified in the code, the scikit-learn defaults are used: Gini impurity, best splitter, minimum samples split 2, and minimum samples leaf 1.	Non-iterative tree fitting; random state 42.	Tree depth limits model complexity.
MLP	Input dimension 24; four hidden fully connected layers with 128 units each; each hidden block uses batch normalization (BN), Rectified Linear Unit (ReLU), and dropout 0.2; final fully connected layer maps to the class number.	Cross-entropy loss; Adam optimizer with learning rate 0.001; batch size 128; 30 epochs in the training function; ReduceLROnPlateau with patience 3 and factor 0.5.	Best checkpoint selected by validation accuracy; dropout and BN used for regularization.
CNN	The 24-dimensional vector is reshaped to 1×4×6. Two convolutional blocks are used: Conv2D 1→32 and 32→32 with 3×3 kernels, BN, ReLU, and max pooling; then Conv2D 32→64 and 64→64 with the same kernel, BN, ReLU, and max pooling. The classifier uses fully connected (FC) layers with dimensions 64–128–64–classes, followed by dropout 0.5 and 0.3.	Cross-entropy loss; Adam optimizer with learning rate 0.001; batch size 128; 30 epochs; ReduceLROnPlateau with patience 3 and factor 0.5.	Best checkpoint selected by validation accuracy; dropout and BN used for regularization.
LSTM	The 24-dimensional vector is reshaped into a pseudo-sequence of length 4 with input size 6. Four unidirectional LSTM layers are stacked, each with hidden size 50, followed by dropout 0.2. The final time step is passed to a fully connected output layer.	Cross-entropy loss; Adam optimizer with learning rate 0.001; batch size 128; 40 epochs in the training function; ReduceLROnPlateau with patience 3 and factor 0.5.	Best checkpoint selected by validation accuracy; dropout used between LSTM layers.

The LSTM is applied to flow-level tabular features by reshaping the selected 24 features into a pseudo-sequential representation. This does not imply that the original flow records are temporal sequences; instead, LSTM is used as an additional nonlinear classifier to test whether the augmented features can improve a sequence-style neural architecture. This limitation is acknowledged when interpreting LSTM results.
